# Advantages and Limitations of Bacteriophages for the Treatment of Bacterial Infections

**DOI:** 10.3389/fphar.2019.00513

**Published:** 2019-05-08

**Authors:** Nicola Principi, Ettore Silvestri, Susanna Esposito

**Affiliations:** ^1^Professor Emeritus of Pediatrics, Università degli Studi di Milano, Milan, Italy; ^2^Department of Surgical and Biomedical Sciences, Pediatric Clinic, Università degli Studi di Perugia, Perugia, Italy

**Keywords:** antibiotics, antimicrobial resistance, bacterial infection, bacteriophages, multi-drug resistance

## Abstract

Bacteriophages (BPs) are viruses that can infect and kill bacteria without any negative effect on human or animal cells. For this reason, it is supposed that they can be used, alone or in combination with antibiotics, to treat bacterial infections. In this narrative review, the advantages and limitations of BPs for use in humans will be discussed. PubMed was used to search for all of the studies published from January 2008 to December 2018 using the key words: “BPs” or “phages” and “bacterial infection” or “antibiotic” or “infectious diseases.” More than 100 articles were found, but only those published in English or providing evidence-based data were included in the evaluation. Literature review showed that the rapid rise of multi-drug-resistant bacteria worldwide coupled with a decline in the development and production of novel antibacterial agents have led scientists to consider BPs for treatment of bacterial infection. Use of BPs to overcome the problem of increasing bacterial resistance to antibiotics is attractive, and some research data seem to indicate that it might be a rational measure. However, present knowledge seems insufficient to allow the use of BPs for this purpose. To date, the problem of how to prepare the formulations for clinical use and how to avoid or limit the risk of emergence of bacterial resistance through the transmission of genetic material are not completely solved problems. Further studies specifically devoted to solve these problems are needed before BPs can be used in humans.

## Introduction

Bacteriophages (BPs) are viruses that can infect and kill bacteria without any negative effect on human or animal cells. For this reason, it is supposed that they can be used, alone or in combination with antibiotics, to treat bacterial infections (Domingo-Calap and Delgado-Martínez, 2018). Administration of BPs for this purpose dates to about a century ago, mainly based on the studies of a French researcher, Felix d’Herelle. Due to his collaboration with his Georgian colleagues, BP therapy was largely used in the Soviet Union in patients of any age suffering from a wide range of diseases. The results were considered very satisfactory and were published in several reports (d’Herelle, 1931; Summers, 2001). However, most of these publications were written in Russian and did not reach the Western world. Moreover, when some results were translated and diffused among English-speaking scientists, they were seen with skepticism, as most of the clinical trials did not follow the international standards (Chanishvili, 2001). The increasing availability of safe and effective antimicrobial drugs after the Second World War has further contributed to the low esteem in which BPs were held in the West until the 1980s (Chanishvili, 2016). However, mainly because most antibiotics were not available, BPs continued to be used in Russia and in Eastern Europe, particularly in those countries previously included in the Soviet Union (Abedon et al., 2011).

The situation has radically changed in the last 30 years, especially in the last decade, as a result of the rapid rise of multi-drug-resistant bacteria worldwide coupled with a decline in the development and production of novel antibacterial agents (Perros, 2015; World Health Organization, 2018). The difficulties in the treatment of many life-threatening bacterial infections have led scientists to reconsider BPs. Several studies regarding BP use *in vitro*, in experimental animals and in humans have been performed in both the United States and Europe. The new interest in BPs is exemplified by their wide use in food production and cattle raising and, regarding therapy in humans, by the activation of a project funded by the European Commission to evaluate phage cocktails to treat burn injuries infected by *Escherichia coli* and *Pseudomonas aeruginosa* (European Commission, 2018) and by the existence in the ClinicalTrials.gov website of 38 clinical studies on a wide range of diseases and conditions in which BPs are tested (U.S. National Library of Medicine, 2018). However, several problems concerning the use of BP to treat infections are not solved, and this explains why no preparation for human use has been licensed by the European Medicine Agency or the Food and Drug Administration. In this narrative review, the advantages and limitations of BPs for use in humans will be discussed. PubMed was used to search for all of the studies published from January 2008 to December 2018 using the key words: “BPs” or “phages” and “bacterial infection” or “antibiotic” or “infectious diseases”. More than 100 articles were found, but only those published in English or providing evidence-based data were included in the evaluation.

## Biology of Bacteriophages (BPs)

Bacteriophages are the most common biological entity. They can be found in soil and seawater, oceanic and terrestrial surfaces and extreme environments, such as those characterized by very high or very low temperatures. Moreover, they have been detected in hospitals, in wastewater and where bacteria can live, including animal and human tissues (Clokie et al., 2011). Several thousand BPs have been described. They have been classified according to their morphological characteristics, their nucleic acid content, the site where they can mostly be found, and the bacterial species that they can kill (Table 1).

**TABLE 1:** Parameters considered in bacteriophages (BPs) for ther classification.

**Parameter**
Biological cycle (lytic or virulent, lysogenic, or temperate)
Morphological characteristics
Nucleic acid content
Site where they can mostly be found
Bacterial target

The most frequently used classification divides tail components of BPs according to their biological cycle (Fauquet and Pringle, 2000; Van Regenmortel, 2007). Two groups are identified, lytic or virulent and lysogenic or temperate BPs. Their biological cycle implies attachment to and invasion of the bacterium. However, to initiate binding, BP structures have to match strain-specific variants of bacterial receptors. Considering the large and continuous mutations of both BP and bacterial structures, frequently mediated by BPs themselves, a single BP can infect a limited number of bacterial strains and, in several cases, only a single strain (Young, 2013). This explains the absolute specificity of BP activity. Once the BP enters the cell, the bacterial synthetic machinery is redirected to the production of viral genome and proteins. Finally, assembly and packing of BPs occurs, and cells are lysed with the release of new virions that can infect other bacterial cells (Young, 2013). However, the burst size can significantly vary according to the phage characteristics, the pathogens against which the BP is directed, and the environments in which the BP-pathogen relationship occurs (Weinbauer, 2004). In contrast, the lysogenic cycle is characterized by integration of viral genetic material into the host genome and, when cell divides, the transmission of virus chromosomes to daughter cells. Only exceptionally can the viral genome be detached from the bacterial DNA and enter the lytic stage (Salmond and Fineran, 2015). Due to their specific ability to kill bacteria, only lytic BPs are used to treat bacterial infections.

## Potential Advantage of Bacteriophage (BP) Use to Treat Bacterial Infections

Theoretically, there are no bacteria that cannot be lysed by at least one BP. In this regard, BPs are significantly more effective than antibiotics, as, although some antimicrobial drugs have a very large spectrum of activity, an antibiotic able to kill all the bacterial species does not exist. However, the most attractive characteristic of BPs is their specificity of action, i.e., their ability to kill only the pathogen that they can recognize.

They have a very narrow spectrum of activity, which avoids the most important problem strictly related to the antibiotic administration, i.e., the influence on the entire microbiome with elimination of potentially beneficial bacteria, the overgrowth of secondary pathogens and the emergence of resistant bacteria (Domingo-Calap and Delgado-Martínez, 2018). Use of BPs without modification of the microbiota has been reported by several studies in both animals and humans. In mice, oral administration of four T4-like BPs effective against diarrhea-associated *E. coli* did not lead to any collateral damage of non-pathogenic bacteria of the same species (Chibani-Chennoufi et al., 2004). In humans, data confirming the specificity of BP action were shown in the study conducted by Sarker et al. (2012). These authors administered for 2 days an oral cocktail of nine T4-like *E. coli* BPs to 15 healthy adults. After a wash-out of 5 days, even though the given BPs could be detected in the feces of almost all treated subjects, no modification of gut microbiota composition was evidenced.

In comparison to antibiotics, BPs are supposed to have several other advantages. It is thought that BPs are significantly safer and better tolerated, as they replicate only in the target bacterium but cannot infect mammalian cells. This conclusion seems supported by all the experiences gathered in the past in Eastern Europe and all the studies carried out more recently in experimental animals and humans, which have not reported significant adverse events following BP administration (Kakasis and Panitsa, 2018). Moreover, administration is easier, as BPs do not need repeated administrations shortly after one another over several days, as is commonly required for antibiotics because they can remain in the human body for relatively prolonged periods of time, i.e., up to several days (Bogovazova et al., 1991, 1992). In general, very few doses are needed because of the increase in BP concentration in the site of infection after the initial administration. Contrarily to antibiotics, their effect is limited to the site of infection that can be reached, even when bacteria are situated in a body organ or system in which antimicrobials can hardly penetrate. A lytic phage, EC200(PP), active against S242, a fatal neonatal meningitis *E. coli* strain, was evaluated in models of meningitis with 100% fatality. Though low titres of the BP were detected in the central nervous system, treatment 1 and 7 h post-infection rescued 100% of pups (Pouillot et al., 2012).

Using the new cost-effective, large-scale DNA sequencing and DNA synthesis technologies, BPs can be engineered to be able to overcome some limitations of antibiotic treatment. A good example of this is given by the evidence that BPs can disperse biofilm, a structure that makes infections difficult to eradicate with standard antibiotic therapy even if bacteria are sensitive to the administered drug. In an *in vitro* study, Lu and Collins engineered a BP affective against an *E. coli* producing biofilm to express a biofilm-degrading enzyme (Lu and Collins, 2007). A simultaneous attack to the bacterial cells and the biofilm matrix was possible. The results were very encouraging, as the engineered BP reduced bacterial biofilm cell count by approximately 99.9%. Moreover, BP genetic modifications can help to fight bacterial resistance to antibiotics. Edgar et al. (2012) introduced in lysogenic phages the genes *rpsL* and *gyrA*, which confer sensitivity in a dominant fashion to two antibiotics, streptomycin and nalidixic acid, respectively. They found that, after engineering, the minimal inhibitory concentrations of bacterial strains previously defined resistant to these drugs were significantly reduced to levels usually found in sensitive pathogens.

Finally, the use of BPs might be less expensive than that of antibiotics whose targets are multidrug-resistant pathogens. In a small group of patients suffering from methicillin-resistant *Staphylococcus aureus* infection, Miedzybrodzki et al. (2007) found that use of BPs significantly reduced healthcare costs.

Table 2 summarizes the main advantages of BPs in comparison to antibiotics.

**TABLE 2:** Potential advantages of BPs in comparison to antibiotics.

**Advantage**
Specificity of action
Narrow spectrum of activity
Higher safety
Higher tolerability
Easy administration
Effect limited to the site of infection
Possible additional benefits after engineering
Less expensive

## Use of Bacteriophages (BPs) for Therapy

Table 3 shows the potential clinical applications of BPs observed in studies carried out in animals and humans.

**TABLE 3:** Potential clinical applications of BPs observed in studies carried out in animals and humans (references in brackets).

**Studies in animals**
Prevention and treatment of *Escherichia coli* acute diarrhea (Smith and Huggins, 1982, 1983; Smith et al., 1987a, 1987b)
Treatment of experimental *Acinetobacter baumanii*, *Pseudomonas aeruginosa*, and *Staphylococcus aureus* infections (Soothill, 1992)
Prevention of skin grafts due to *Pseudomonas aeruginosa* (Soothill, 1994)
Reduction of intestinal colonization of food-producing animals by *Escherichia coli*, *Campylobacter jejuni*, and *Salmonella* spp (Rosenquist et al., 2003; Loc Carrillo et al., 2005; Sheng et al., 2006; Carvalho et al., 2010)
Treatment of chronic, refractory *Pseudomonas aeruginosa* otitis media (Hawkins et al., 2010)
Reduction of disease severity in *Escherichia coli* and *Pseudomonas aeruginosa* respiratory infection (Huff et al., 2003; Carmody et al., 2010; Morello et al., 2011; Alemayehu et al., 2012; Singla et al., 2015)

**Studies in humans**
Topical treatment of skin bacterial infections (mainly due to *Pseudomonas aeruginosa* or *Staphylococcus aureus* (Abul-Hassan et al., 1990; Markoishvili et al., 2002; Rhoads et al., 2009; Kvachadze et al., 2011; Rose et al., 2014; Fish et al., 2016, 2018; Jault et al., 2018; Morozova et al., 2018a, 2018b; Rohde et al., 2018)
Treatment of cholera (Summers, 1993)
Treatment of chronic, refractory *Pseudomonas aeruginosa* otitis media or otitis externa (Essoh et al., 2013)
Lung infection due to *Pseudomonas aeruginosa* or *Achromobacter xylosoxidans* in cystic fibrosis patients (Kutateladze and Adamia, 2008, 2010; Hoyle et al., 2018)

### Studies in Animals

The most important studies of BPs carried out in animals in the Western world date back to the 1980s, when Smith et al. made several attempts to evaluate whether the use of specific BPs against *E. coli* could prevent and treat acute diarrhea due to these pathogens (Smith and Huggins, 1982, 1983; Smith et al., 1987a, b). The results were encouraging, as infections due to oral inoculation of a mixture of 6 pathogenic *E. coli* strains were controlled by the administration of a single dose of a 10^5^ pool of the six BPs active against them. A lower dose (10^2^) was enough to prevent diarrhea. Further experimental evidence of the benefits deriving from the use of BPs was reported some years later by Soothill, who showed that specific BPs could control experimental *Acinetobacter baumanii, P. aeruginosa*, and *S. aureus* infections in mice (Soothill, 1992). Moreover, the same authors showed that BP administration prevented the destruction of skin grafts by *P. aeruginosa* in guinea pigs (Soothill, 1994). More recent studies have found that BP treatment of food-producing animals is associated with a reduction of the risk of contamination of the resulting food products during processing (Rosenquist et al., 2003; Loc Carrillo et al., 2005; Sheng et al., 2006; Carvalho et al., 2010). A 1- or 2-log reduction in the number of pathogens shed in feces of the slaughtered animals could reduce the risk by 45–75%, respectively. Moreover, several reports of reduced intestinal colonization of food-producing chickens and ruminants by *E. coli, Campylobacter jejuni*, and *Salmonella* spp. after BP administration have been published, and this has led to the wide use of BPs to prevent food contamination (Rosenquist et al., 2003; Loc Carrillo et al., 2005; Sheng et al., 2006; Carvalho et al., 2010).

Finally, several attempts to use BPs to treat respiratory infections of animals have been made. For instance, Hawkins et al. (2010) used BPs to treat 10 dogs with chronic, refractory *P. aeruginosa* otitis media. In each animal, a single dose of a topical preparation containing approximately 1 × 10^5^ plaque-forming units (PFU) of each of 6 BPs, specific for these pathogens, was instilled directly into the auditory canal of one ear. Before therapy and 48 h later, conditions of the affected ear were assessed through the evaluation of a clinical score based on 5 indicators, and aural swabs were taken to measure BP and *P. aeruginosa* concentrations. Treatment was very effective and safe. Mean score was reduced by 30.1% (range 7.7–56.3%, *p* < 0.0001), and pathogen mean count declined by 67% [95% confidence interval (CI) 52–82, *p* < 0.001]. Improvement was accompanied by a significant increase in BP/swab counts (mean 5.9 × 10^7^ PFU/swab, range 1.7 × 10^6^ to 2.6 × 10^8^ PFU/swab). In all the animals, BP counts were higher than those administered (mean rise: 99.1-fold, range 2.8–433.3-fold), showing significant viral replication. Treatment did not cause related inflammation or other adverse events, either local or systemic. Moreover, administration of BPs with an aerosol spray reduced the mortality of broiler chickens with *E. coli* respiratory infection (Huff et al., 2003) and limited the clinical problems of mice infected with *P. aeruginosa* (Morello et al., 2011; Alemayehu et al., 2012), *Klebsiella pneumoniae* (Singla et al., 2015), or *Burkholderia cenocepacia* (Carmody et al., 2010).

### Studies in Humans

Bacteriophages have been used to treat bacterial infections involving different body sites with various preparations. The greatest number of these studies have investigated the use of BPs for topical treatment of skin bacterial infections. Beneficial effects of BP administration for treatment of localized infections in wounds, burns, and trophic ulcers, including diabetic foot ulcers, were reported during and after the Second World War in Eastern Europe (Morozova et al., 2018b). Similar results were later shown by a number of studies published in Russian with abstracts in English (Rohde et al., 2018) and finally confirmed, although with a few exceptions (Rose et al., 2014), by a series of trials carried out in the Western world and published in English in peer-reviewed journals (Markoishvili et al., 2002; Rhoads et al., 2009; Kvachadze et al., 2011; Fish et al., 2016, 2018; Jault et al., 2018; Morozova et al., 2018a).

The topical application of BPs cured burns infected by multidrug-resistant *P. aeruginosa* and allowed successful skin grafting in 18 out of 30 adult patients in whom this therapy was used (Abul-Hassan et al., 1990). Positive results were reported in several studies that used BPs in patients with infected ulcers (Rhoads et al., 2009; Fish et al., 2016, 2018; Morozova et al., 2018a), especially when BPs were included in a biodegradable wound dressing that released active antimicrobials, including BPs, for a long time (Kvachadze et al., 2011). For instance, patients with diabetic foot ulcers infected by *S. aureus* strains benefited from the administration of the *Staphylococcus* BP Sb-1 (Jault et al., 2018), even when BP administration therapy was the only antimicrobial treatment (Fish et al., 2018; Morozova et al., 2018b). Other BPs, specific for different bacterial pathogens, were effective in a number of diabetic foot ulcer infections previously treated unsuccessfully with antibiotics (Rhoads et al., 2009; Fish et al., 2016, 2018; Morozova et al., 2018a).

However, the positive effects of topical use of BPs to treat bacterial skin infections are partly questioned by the results of the study by Jault et al. (2018) the only randomized clinical trial carried out to evaluate impact of BPs on skin infections. These authors randomly assigned adult patients with a burn wound clinically infected with *P. aeruginosa* to receive a cocktail of 12 BPs (PP1131; 1 × 10^6^ PFU/mL) or standard of care (1% sulfadiazine silver emulsion cream), both administered topically, once a day for 7 days, with 14 days of follow-up. Thirteen patients received BPs and 14 the standard therapy. Unfortunately, although BPs had an effect, the primary endpoint of the study, i.e., the median time to sustained reduction of bacterial burden, was reached significantly more slowly with BPs than with sulfadiazine cream (144 h vs. 47 h, *p* = 0.018). This can be explained by the evidence that the BP preparation was not stable, and patients received a 1,000-fold to 10,000-fold lower dose of active BPs than initially prescribed.

Oral administration of BPs was effective in prevention and treatment of cholera, but no randomized controlled trials enrolling patients with this disease have been conducted (Summers, 1993). The results on *E. coli* diarrhea have been partially disappointing. Studies carried out in both adults and children have found that 2 BP cocktails specifically active against some *E. coli* strains were safe and well tolerated and could be used to treat diarrhea due to these pathogens (Sarker et al., 2012; McCallin et al., 2013). The first contained eleven T4-like phages (AB2, 4, 6, 11, 46, 50, 55; JS34, 37, 98, D1.4) (Bourdin et al., 2014). The second, a Russian preparation, consisted of at least 17 different phage types, including T7 phage (McCallin et al., 2013). However, a prospective, randomized, placebo-controlled, parallel-group clinical trial carried out in Bangladesh in children aged 6–24 months did not find any positive effect on diarrhea evolution. With both preparations, no amelioration in quantitative diarrhea parameters over standard care was shown (Sarker et al., 2016). Moreover, an increase in gut content of *Streptococcus gallolyticus* and *Streptococcus salivarius* species groups strictly related to the quantitative diarrhea outcome was evidenced. These results suggested a potential modification of gut microbiota. The failure was explained by the evidence that, despite a careful selection of cases, only 60% of the 120 enrolled patients harbored an *E. coli* strain in the stool, as well as by the poor replication of BPs, as the BP content of the gut was never higher than the administered doses (Sarker et al., 2016).

Bacteriophages were also used to treat respiratory tract infections. Wright et al. (2009) conducted a randomized, double-blind, placebo-controlled phase I/II clinical trial administering a preparation containing 6 BPs active against *P. aeruginosa* or placebo to a group of 24 adult patients suffering from chronic, refractory aural discharge due to previous mastoid surgery, chronic perforations, or longstanding otitis externa. Only patients with *P. aeruginosa* infection due to strains susceptible to the used BPs were enrolled. BPs or placebo was instilled in the ear canal and each patient was re-examined at days 7, 21, and 42 after treatment. Relative to baseline, clinical improvement was more common among treated patients. Moreover, the deterioration seen in some placebo-receiving subjects was not evidenced in patients given BP. The reduction in *P. aeruginosa* concentration was significantly higher in treated patients than in controls at days 21 and 42 (*p* = 0.009 and *p* = 0.016, respectively), with a strict relationship between the disappearance of the pathogen and improvement in otoscopy. The mean recovery of BP in treated patients at the three study visits exceeded 200 times the input dose. No treatment-related adverse event was reported.

A number of *in vitro* studies have suggested that BPs could be effective in treating cystic fibrosis (CF) patients. Essoh et al. (2013) purified 6 BPs from a Russian cocktail, including 4 genera effective against *P. aeruginosa*, and tested them against the CF sputum. It was shown that 33 out of 47 *P. aeruginosa* strains were lysed. Saussereau et al. (2014) showed that the addition of a cocktail of 10 BPs infecting *P. aeruginosa* to sputum samples of CF patients led to a significant reduction of pathogens in comparison to controls (*p* = 0.024). In 5.8% of the cases, these results were associated with a relevant increase in BP concentration. In both these studies, BP therapy overcame the obstacles of the CF environment, mainly the mucus characteristics and the biofilm inhibition (European Commission, 2018). However, studies in humans are few and limited to case reports. No randomized, placebo-controlled trial is presently available. Single cases of CF patients with chronic, otherwise untreatable, lung infection due to *P. aeruginosa* (Kutateladze and Adamia, 2008) or *Achromobacter xylosoxidans* (Hoyle et al., 2018) that received BPs per aerosol with good results have been described. Moreover, aerosolized BPs were administered to 8 CF patients together with the conventional antibiotic treatment for 6–10 days. Both *P. aeruginosa* concentration in the respiratory secretions and antibiotic need were reduced (Kutateladze and Adamia, 2010). However, definitive conclusions on the relevance of BP treatment in patients with CF cannot be drawn.

## Factors That Can Limit Bacteriophage (BP) Use and Development of New Preparations for Therapy

Data regarding the use of BPs to treat bacterial disease in humans are few, sometimes conflicting or negative and almost always collected in trials that are not randomized and placebo controlled. Moreover, the preparation of BPs for clinical use is difficult, and not all the problems strictly related with BP biology have been solved. Table 4 describes the main limitations associated with BP use.

**TABLE 4:** Main limitations associated with bacteriophage (BP) use.

**Limitation**
Absence of specific activity for a given bacterial strain
Difficulty in production of BP genome without integrase genes, antibiotic resistant genes, genes for phage-encoded toxins or genes for other bacterial virulence factors
Problems related to the formulation and stabilization of pharmaceutical preparations
Possible emergence of bacterial resistance against BPs
Contribution of BPs in the development of antibiotic resistance
Reduced activity due to immune system response to BPs

### Problems Related to the Choice of the BPs Effective for the Various Bacterial Infections

Identification of a therapeutic BP is very complicated. Isolation of BPs, generally from wastewater and sewage, is the first step and is *per se* relatively easy, although with differences between the various bacterial pathogens. For example, it is significantly easier if *P. aeruginosa* rather than *S. aureus* is the target of the BP (Mattila et al., 2015). However, before a BP is identified as a potential therapeutic agent, it has to be demonstrated that it is specific for a given bacterial strain. This is a relatively complicated problem, as evidence of lytic capacity of a BP can vary according to the interrelationships between the BP and bacterium and their modification with time together with the dose of virus used for the test. Moreover, the BP genome must be sequenced and not contain integrase genes, as in the lysogenic type, antibiotic resistant genes (ARG), genes for phage-encoded toxins or genes for other bacterial virulence factors. Finally, problems related to the formulation and stabilization of pharmaceutical preparations for clinical use are far from solved (Vandenheuvel et al., 2015). In this regard, it has to be highlighted that studies seem to indicate that stability of preparations for clinical use is strictly BP dependent and stabilization strategies should be optimized for each BP separately (Clark, 1962; Merabishvili et al., 2013). This can lead to costly and time-consuming clinical trials that could discourage the pharmaceutical industry from starting research and production of preparations for human use.

### The Risk of Emergence of Bacterial Resistant to Bacteriophages (BPs)

Emergence of bacterial resistance against BPs is potentially possible, as bacteria possess or can develop several mechanisms to prevent viral infections. Among these are hiding, change or loss of receptor, secretion of substances that prevent phage adhesion to the bacterial pathogen, activation of measures for blocking phage DNA injection into the cell and inhibition of phage replication and release (Seed, 2015). Alteration or loss of receptor for membrane protein modifications has been demonstrated for *E. coli* (Riede and Eschbach, 1986), *S. aureus* (Nordström and Forsgren, 1974), *Bordetella bronchiseptica* (Liu et al., 2002), and *Vibrio cholerae* (Seed et al., 2012). Secretion of extracellular polymeric substances and glycoconjugates has been described for *Pseudomonas* spp. and *Enterobacteriaceae* (Drulis-Kawa et al., 2012), respectively. Development of bacterial resistance to BPs can be reduced with the use of BP cocktails, with the administration of a higher initial BP inoculum or the association with antibiotics. If BPs kill pathogens faster than they can replicate, a high inoculum is associated with a lower risk of development of BP-resistant bacteria. However, all these findings indicate that selection of a therapeutic BP must take into account the ability of each virus to induce bacterial resistance and the amount needed to avoid bacterial resistance development (Torres-Barceló, 2018a).

### Risk That Bacteriophages (BPs) May Contribute to the Development of Antibiotic Resistance

Lysogenic phages incorporate their DNA into the bacterial genome. Consequently, they might be vehicles for horizontal exchange of genetic material and play a role in the diffusion of antibiotic resistance genes (ARGs). Theoretically, due to transduction, new microbes or even more resistant bacteria can develop (O’Shea and Boyd, 2002; Brabban et al., 2005; Maiques et al., 2007). However, the real contribution of phages to the diffusion of ARGs is not precisely defined. Most of the studies specifically planned to measure how often phages encode ARGs have suggested that these viruses are reservoirs of ARGs. Genes such as *blaTEM* (resistance to β-lactams), *qnrS* (reduced susceptibility to fluoroquinolones), *ermB* (resistance to macrolides), *sulI* (resistance to sulphonamides), and *tetW* (resistance to tetracyclines) have been found in the virome of activated sludge and environmental water samples (Parsley et al., 2010; Marti et al., 2014; Rodriguez-Mozaz et al., 2015; Subirats et al., 2016) and in the phage DNA fraction isolated from the intestinal mucus of wild freshwater fish species (Marti et al., 2018) and human fecal samples (Quirós et al., 2014). Moreover, diffusion of ARGs can be significantly favored by the presence in the environment of phage inducers, i.e., substances able to induce the expression of prophage gene products or lead to the excision and spread of temperate BPs. Some antibiotics, such as fluoroquinolones and some anticoagulants, are the most important (Torres-Barceló, 2018b). Treatment of wastewater samples with EDTA or sodium citrate activates the lytic cycle of lysogenic phages and leads to the generation of new phage particles, bacterial lyses, phage release outside the cell, and infection of a greater number of bacteria (Colomer-Lluch et al., 2014).

Finally, great numbers of phages carrying genes associated with antibiotic resistance have been detected in secretions and tissues of patients who suffer from recurrent infections due to antibiotic resistant pathogens and had been previously repeatedly treated with antimicrobial drugs. One of the best examples in this regard is CF, as evidenced by the study conducted by Fancello et al. (2011). These authors analyzed 1,031 short sequences in the CF virome putatively encoding antibiotic resistance and identified 66 efflux pump genes, 15 fluoroquinolone resistance genes and 9 β-lactamase genes. This and similar findings led to the supposition that BPs might be vehicles for the adaptation of bacteria to the CF lung environment and for the emergence and selection of multidrug-resistant pathogens with chimeric repertoires (Rolain et al., 2011). However, a recent reevaluation of previously collected data has suggested that AGR abundance in phages was vastly overestimated and that the risk of transduction, although possible, is lower than previously thought. In several studies, conclusions were misled by the excessive bacterial DNA content of the studied samples. Moreover, inadequate approaches to detect ARGs in phage genomes have been used (Enault et al., 2017). Partially in agreement with these findings were Lekunberri et al. (2017) who carried out an analysis of 33 viromes from human feces, pig feces, raw sewage, and freshwater and marine environments . The human-associated viromes did not carry or rarely carried ARGs, while those deriving from non-human sources harbored a significantly higher prevalence of ARGs.

### Reduced Activity Due to Immune System Response

Bacteriophages and their products are non-self-antigens, and it is not surprising that they can be recognized by the immune system and induce responses that can theoretically reduce the benefit of BP administration. Immune response to BPs has been demonstrated in both experimental animals and humans, although with differences according to the phage strain, the route of administration and the prior exposure. In animals, BPs were taken up by phagocytic cells a few minutes after the administration and might be destroyed by these cells within 2 h (Kaźmierczak et al., 2014). Moreover, examining the survival of T7 BP in the blood of healthy and immunocompromised mice, it was shown that, whereas in animals with severe combined immunodeficiency, BP titres were stable for a long time, in healthy mice 99% of BPs were eliminated within 60 min of the injection. As phage titres remained stable in the B-cell-deficient mouse, the greatest part of phage clearance from blood seemed to be due to specific antibody production (Srivastava et al., 2004).

In humans, Dąbrowska et al. (2014) who have studied the antigenicity of the proteins forming the *E. coli* T4 BP head surface, have reported that specific antibodies could be detected in more than 80% of enrolled individuals, although none of them had received phage therapy . However, even if not fully demonstrated, it seems likely that the immune response evoked by phages has a marginal or no impact on the potential bacterial killing of phage administration. Bacterial lysis occurs before a specific antibody is evoked. Moreover, with some exceptions, phage administration was generally not associated with tissue damage, an increase in pro-inflammatory cytokines or increased reactive oxygen species (ROS) production (Park et al., 2014). Miernikiewicz et al. (2013) have shown that phage T4 and its head proteins given intraperitoneally to mice did not affect the production of cytokines [interleukin (IL)-1α, IL-1β, IL-2, IL-6, IL-10, IL-12 p40/p70, interferon (IFN)-γ, tumour necrosis factor (TNF)-α, monocyte chemoattractant protein (MCP-1), monokine induced by gamma (MIG), RANTES, granulocyte colony-stimulating factor (GCSF), and granulocyte-macrophage colony-stimulating factor (GM-CSF)] or ROS. Similar findings were reported by Hwang et al. (2016) who studied the safety of an *E. coli* phage cocktail given by mouth to rats for 4 weeks. Carmody et al. (2010) studied the efficacy of phage therapy given by intranasal inhalation in a mouse model of *B. cenocepacia* pulmonary infection. Bacterial density, macrophage inflammatory protein 2 (MIP-2), and TNF-α were not increased but, on the contrary, were significantly reduced in lungs of treated mice compared to untreated controls (*p* < 0.05). On the other hand, data collected in humans that seem to indicate that BP administration is safe seem to support that, even if present, the immune response to BPs is not clinically important.

## Conclusion

Use of BPs to overcome the problem of increasing microbial resistance to antibiotics is attractive, and some research data seem to indicate that it might be a rational measure. However, present knowledge seems insufficient to allow the use of BPs for this purpose. To date, properly designed clinical trials specifically planned to evaluate BP efficacy are very few and partially negative. Moreover, the problem of how to prepare the formulations for standardized and clinical use in bacterial control, how to avoid or limit the risk of emergence of bacterial resistance and the transmission of genetic material are not completely solved problems. In addition, the mechanisms concerning coevolution between BP and bacteria are unknown. Further studies specifically devoted to solve these problems are needed before BPs can be used in humans.

## Author Contributions

NP and SE contributed to writing the manuscript. ES performed the literature review. All authors approved the final submitted version of the manuscript.

## Conflict of Interest Statement

The authors declare that the research was conducted in the absence of any commercial or financial relationships that could be construed as a potential conflict of interest.
